# Estrogen receptor and temperature independently influence sex determination in the red-eared slider turtle

**DOI:** 10.3389/fendo.2025.1632672

**Published:** 2025-07-21

**Authors:** Xifeng Wang, Zihan Ding, Pengfei Wu, Jiong Fu, Weiguo Du

**Affiliations:** ^1^ Key Laboratory of Animal Ecology and Conservation Biology, Institute of Zoology, Chinese Academy of Sciences, Beijing, China; ^2^ University of Chinese Academy of Sciences, Beijing, China; ^3^ School of Life Sciences, Fudan University, Shanghai, China

**Keywords:** red-eared slider turtle, temperature-dependent sex determination (TSD), estrogen, estrogen receptor (ESR), ovarian differentiation

## Abstract

In reptiles with temperature-dependent sex determination (TSD), including the red-eared slider turtle *Trachemys scripta elegans*, female sex determination is sensitive to estrogen. However, the underlying molecular mechanism by which estrogen facilitates ovarian development remains unclear in TSD. Here, we explore the role of estrogen receptors (ESRs) in ovarian differentiation by administering 17β-estradiol (E2), as well as agonists and antagonists of ESRs to embryos of red-eared sliders. We found that treatment with E2 or one of the ESR (ESRα, ESRβ, or GPER1) agonists induced typical female characteristics of gonads at the male-producing temperature (MPT), exhibiting advanced outer cortex and degraded medullary cord as well as upregulation of *Cyp19a1* and *Foxl2* and downregulation of *Amh* and *Dmrt1*. In addition, this male-to-female sex reversal induced by E2 at MPT can be reversed by using a combination of three ESR antagonists. However, antagonizing any of the three ESRs or the three ESRs together did not affect ovarian differentiation at the female-producing temperature (FPT). Our study demonstrates that estrogen regulates the expression of estrogen-responsive sex-specific genes through the ESRs to induce ovarian differentiation at MPT, and ESRs do not have to engage in ovarian development directly at FPT, indicating that alternative pathways might drive feminization under natural high-temperature conditions.

## 1 Introduction

Traditional studies on sex determination mainly focused on testis development rather than ovarian development, because ovarian development had long been considered a “default” developmental outcome switched on passively by the absence of sex-determining region Y (SRY) in mammals (1, 2). Recently, increasing lines of evidence demonstrate that active mechanisms are required to produce an ovary in vertebrates (3–6). In many reptiles, including the red-eared slider turtle (*Trachemys scripta elegans*) with temperature-dependent sex determination (TSD), female sex determination is sensitive to both temperature and estrogen during the temperature-sensitive period (TSP) (7–9). Warmer temperature can affect the expression of sex-related genes, which induce ovarian determination and differentiation in *T*. *scripta* (6, 10). Estrogen, a major steroid hormone, plays an essential role in ovarian development in vertebrates (11–14). It is not only critical for the development of female secondary sexual characteristics and female reproduction in eutherian mammals, but also essential to female sex determination and differentiation in non-eutherian vertebrates (11–16). Additionally, a synergistic effect between warm temperature and estrogen action can be seen in turtle sex determination—less estrogen is required to sex-reverse embryos from intermediate temperatures than from extreme male-producing temperatures (MPTs) (17, 18). Indeed, the administration of estrogen/aromatase inhibitor can influence sex determination while the ambient temperature during the TSP of embryo development primarily determines the sex of offspring (19–23). For example, estrogen treatment caused premature suppression of a central male sex-determining gene, which is indispensable for testis differentiation, SRY-box transcription factor 9 (SOX9), and the dissolution of cord structures in the medulla, while aromatase inhibition maintained SOX9 and testis cords, and resulted in ovotestis development in *T*. *scripta* (24–26). Nevertheless, the precise molecular mechanism underlying estrogen action on gonadal development in TSD reptiles remains poorly understood.

This mechanism may involve estrogen receptors (ESRs) since estrogen mediates its actions by binding to the two classical nuclear ESRs (ESRα/ESR1 and ESRβ/ESR2) to regulate target gene transcription or by activating the G protein-coupled estrogen receptor 1 (GPER1/GPR30) to elicit downstream signaling cascades (27, 28). These receptors are found to participate in regulating ovarian function in several vertebrates regardless of the mode of sex determination (29–33), but the involvement of specific ESR differs among lineages. For example, ESRα and ESRβ are necessary for normal ovarian function but have no gross effect on ovarian differentiation in mammals (29, 34, 35). ESRα plays an important role in the differentiation of reproductive tracts in female embryos of birds and reptiles (30, 31, 36–39), while ESRβ is more essential for ovary development in bony fish (33, 38, 40). Additionally, GPER1 is not required for ovarian differentiation or function in mammals, birds, and fish (41, 42). Therefore, verifying the working ESRs on TSD not only is important for our understanding of how estrogen affects ovarian differentiation in the species of interest but also provides new insights into the role of ESR in sex determination of vertebrates.

To investigate the precise molecular mechanism underlying the action of estrogen–ESR on ovarian differentiation in TSD species, we conducted a series of experiments in the *T. scripta* following a female–male TSD pattern, of which eggs incubated at warm temperature (31°C) produces all females, whereas cold temperature (26°C) produces all males (10, 43). We administered 17β-estradiol (E2) and specific pharmaceutical agonists and antagonists for ESRs (ESRα, ESRβ, and GPER1) to embryos of *T. scripta* at MPT or FPT during TSP. Subsequently, we performed immunocytochemical analysis and real-time quantitative PCR (RT-PCR) to examine gonadal histology and specific sex gene expression patterns. Our results demonstrated that agonists of ESRα, ESRβ, or GPER1 can induce sex-reversed females at MPT, accompanied by the upregulation of female-related sex-determining genes and the downregulation of male-related sex-determining genes. Moreover, this male-to-female sex reversal induced by E2 at MPT can be reversed by blocking the three ESRs together. In contrast, antagonizing one or three ESRs did not affect ovarian differentiation of turtle embryos incubated at FPT. These findings indicate that estrogen regulates the expression of estrogen-responsive sex-related genes through the three ESRs—ESRα, ESRβ, and GPER1—to induce ovarian differentiation of *T*. *scripta* at MPT, whereas warmer temperatures trigger female-specific regulator expression underpinning ovarian development of *T*. *scripta* at FPT without ESRs’ engagement directly.

## 2 Materials and methods

### 2.1 Egg incubation and tissue collection

We obtained freshly laid eggs (within 1 day of being laid) of *T. scripta* from a turtle farm in Hanshou (Hunan, China). Fertilized eggs were collected and rinsed in distilled water to clean the outer shell and then placed in small plastic boxes containing a 1:1 (by mass) vermiculite:water mixture and incubated at either 26°C (MPT) or 31°C (FPT) using two KB240 thermal-regulation systems (Binder, Germany). Incubation at these temperatures yields 100% male and 100% female embryos, respectively (9, 44). The progress of development was monitored by dissection of one to two eggs at regular intervals. Embryos were staged according to criteria established by Greenbaum (45). Eggs were opened and embryos were immediately decapitated and placed into phosphate-buffered saline (PBS) for gonad–mesonephros complexes (GMCs) at stage 25 and whole-gonad collection at different developmental stages (including stage 16, stage 17, stage 18, stage 19, stage 20, stage 21, and stage 25) (46). All experiments were carried out under the guidelines specified by the Animal Care and Use Committee at the Institute of Zoology, Chinese Academy of Sciences (IOZ-IACUC-2023-152).

### 2.2 Chemical treatments

We dissolved E2 (17β-estradiol) (Sigma-Aldrich) in ethanol at a final concentration of 1.0 μg/μL and we dissolved the estrogenic compounds in dimethyl sulfoxide (DMSO) (Sigma-Aldrich) with the following concentrations: 10.0 μg/μL of ESRα-specific agonist PPT (4,4,4’-(4-propyl-[1H]-pyrazole-1,3,5-triyl) tris-phenol) (MedChemExpress), 10.0 μg/μL of ESRα-specific antagonist AZD9496(3-[3,5-difluoro-4-[(1r,3r)-2-(2-fluoro-2-methylpropyl)-2,3,4,9-tetrahydro-3-methyl-1h-pyrido[3,4-b]indol-1-yl] phenyl]-2-propenoic acid) (MedChemExpress), 10.0 μg/μL of ESRβ-specific agonist WAY20007 (7-bromo-2-(4-hydroxyphenyl)-1,3-benzoxazol-5-ol) (MedChemExpress), 10.0 μg/μL of ESRβ-specific antagonist PHTPP (4-[2-phenyl-5,7-bis (trifluoromethyl) pyrazolo [1,5-a] pyrimidine-3-yl] phenol) (MedChemExpress), 0.5 μg/μL of GPER-specific agonist G1 (1-[(3aR,4S,9bS)-4-(6-bromo-1,3-benzodioxol-5-yl)-3a,4,5,9b-tetrahydro-3H cyclopenta [c] quinolin-8-yl]) (MedChemExpress), and 0.5 μg/μL of GPER-specific antagonist G15 ((3aR,4R,9bS)-4-(6-bromo-1,3-benzodioxol-5-yl)-3a,4,5,9b-tetrahydro-3H-cyclopenta[c] quinolone) (MedChemExpress).

When the embryos were estimated to be approaching stage 16 (within the thermo-sensitive period), a total 1,350 eggs were randomly assigned to nine treatment groups, with 150 eggs in each chemical treatment group. We wiped the end of eggs with alcohol and punched a small hole with a needle. We injected solvent with the active chemical through the hole in the shell into the turtle eggs with a fine metal needle (Hamilton Company, USA, size: 0.5 mm) and classified them into the following groups (see **Supplementary Table S1**): MPT + E2 (eggs with 0.5 μg/g of E2 at MPT), MPT + PPT (eggs with 5 μg/g of PPT at MPT), MPT + WAY200070 (eggs with 5 μg/g of WAY200070 at MPT), MPT + G1 (eggs with 0.25 μg/g of G1 at MPT), FPT + AZD9496 (eggs with 5 μg/g of AZD9496 at FPT), FPT + PHTPP (eggs with 5 μg/g of PHTPP at FPT), FPT + G15 (eggs with 0.25 μg/g of at FPT), MPT + E2 + Tripo (eggs with 0.5 μg/g of E2, 5 μg/g of AZD9496, 5 μg/g of PHTPP, and 0.25 μg of G15 at MPT). The control group was treated with the corresponding solvent without the active chemical.

### 2.3 Gonadal histology

GAMs from embryos were washed three times with PBS and then were observed with a stereomicroscope (Leica, Germany), and pictures were obtained digitally. Fresh dissected gonads were fixed in 4% (w/vol) paraformaldehyde (PFA) at 4°C overnight. On the second day, fixed GAMs were paraffin embedded for histologic analyses. Serial 5-μm sections of the gonads were stained with hematoxylin and eosin (H&E) by a standard procedure for microscopic examination and assignment of sex. Three independent blinded investigators determined the phenotypic features of the gonads.

### 2.4 RNA extraction and quantitative RT-PCR

Gonads at different developmental stages (stage 16, stage 17, stage 18, stage 19, stage 20, stage 21, and stage 25) from 15 individual embryos in each group were harvested for RNA extraction using the RNeasy Plus Micro kit (Qiagen) following the manufacturer’s instructions. The quantity and quality of the RNA were assessed using the NanoDrop 2000 (Thermo Scientific, USA) and by gel electrophoresis. A two-step RT-PCR analysis approach was taken. RNA (0.5–2 μg) was reverse transcribed using the HiFiScript cDNA Synthesis Kit (CWBIO) following the manufacturer’s instructions. Real-time amplification was performed on a LightCycler^®^ 480II real-time PCR system (Roche, Germany), using the manufacturer’s recommended program for ChamQ Universal SYBR qPCR Master Mix kit (Vazyme) in a standard 96-well block. Reactions were conducted in 20-μL volumes containing 2 × SYBR Premix Taq, 1 μL of first-strand cDNA, and 5 pmol of each primer. Thermal cycling conditions consisted of 1 cycle of 95°C for 3 min and 40 cycles of denaturation at 95°C for 10 s and annealing/extension at 60°C for 20 s. We measured the expression levels of *ESRα*, *ESRβ*, and *GPER1* in embryonic gonads from stage 16 to stage 21, and stage 25 of embryonic development at both FPT and MPT, and also measured the expression levels of cytochrome P450 family 19 subfamily A member 1 (*Cyp19a1*), *forkhead box L2* (*Foxl2*), anti-Mullerian hormone (*Amh*), and doublesex and mab-3 related transcription factor 1 (*Dmrt1*) in embryonic gonads from stage 25 in each chemical treatment group and control group. Analysis of the results was performed employing a comparative Ct method with the gene glyceraldehyde-3-phosphate dehydrogenase (*Gapdh*) as the endogenous control (47). For each assay, three biological replicates and three technical replicates were performed. The sequence of primers for RT-PCR is listed in **Supplementary Table S2**.

### 2.5 Immunohistochemistry and confocal imaging

Freshly dissected gonads were fixed in 4% (w/vol) PFA in PBS for 2 h at 4°C. After washing with increasing concentrations of methanol, tissues were embedded in paraffin wax and sliced into 5-μm serial sections (Carl Zeiss, Germany). Deparaffinized sections of the gonads were immersed in antigen retrieval buffer (10 mM/L citrate buffer, pH 6.0) at 95°C for 10 min and washed twice in PBS with 0.01% Tween-20, then incubated for 1 h at room temperature in a blocking solution consisting of 10% heat-inactivated fetal bovine serum (FBS), 3% bovine serum albumin (BSA), and 0.2% Triton X-100 in PBS followed by overnight incubation at 4°C in primary antibodies diluted in the blocking solution. Sections were rinsed three times in PBS and incubated in secondary antibodies in the blocking solution for 1 h at room temperature. A Zeiss 710 inverted confocal microscope (Carl Zeiss, Germany) was used to collect images, and the Zeiss free offline software was used to generate maximum intensity projections.

Primary antibodies were goat anti-Foxl2 (privately produced) used at 1:250; rabbit anti-Sox9 (AB5535, Chemicon, USA) used at 1:1,000; rabbit anti-Vasa (ab13840, Abcam, CA) used at 1:200; and mouse anti-β-catenin (C7207, Sigma, USA) used at 1:250. Secondary antibodies were Alexa Fluor 594 donkey anti-rabbit IgG (A21207, Invitrogen, USA), Alexa Fluor 594 donkey anti-mouse IgG (A21203, Invitrogen, USA), Alexa Fluor 488 donkey anti-rabbit IgG (A21206, Invitrogen, USA), and Alexa Fluor 488 donkey anti-mouse IgG (A21202, Invitrogen, USA), all of which were used at 1:250 to detect primary antibodies. Cell nuclei were stained with DAPI (C1006, Beyotime, China).

### 2.6 Statistical analyses

Data were visualized and analyzed using GraphPad Prism 8 (GraphPad Software, San Diego, CA, USA) and R (version 4.1.0) (48). The effects of chemical treatments on sex ratio were tested using the chi-square test. The Student’s unpaired *t*-test was employed to test the differences in *Dmrt1*, *Amh*, *Foxl2*, and *Cyp19a1* gene expression between the treatment and the control group, as well as the differences in gene expression of *ESRα*, *ESRβ*, and *GPER1* at different incubation temperatures during the same stage. We utilized the Kruskal–Wallis test and Dunn’s comparison to analyze the differences in gene expression at each developmental stage of ESRs under MPT or FPT, as well as three ESRs’ expression across different developmental stages under both MPT and FPT conditions. Each experiment was independently repeated at least three times. All data are presented as means ± standard error of the mean (SEM). Significant effects were considered at *p* ≤ 0.05.

## 3 Results

### 3.1 Expression profile of ESRα, ESRβ, and GPER1 during development of gonads in *T. scripta* at MPT and FPT

**Figure 1:**
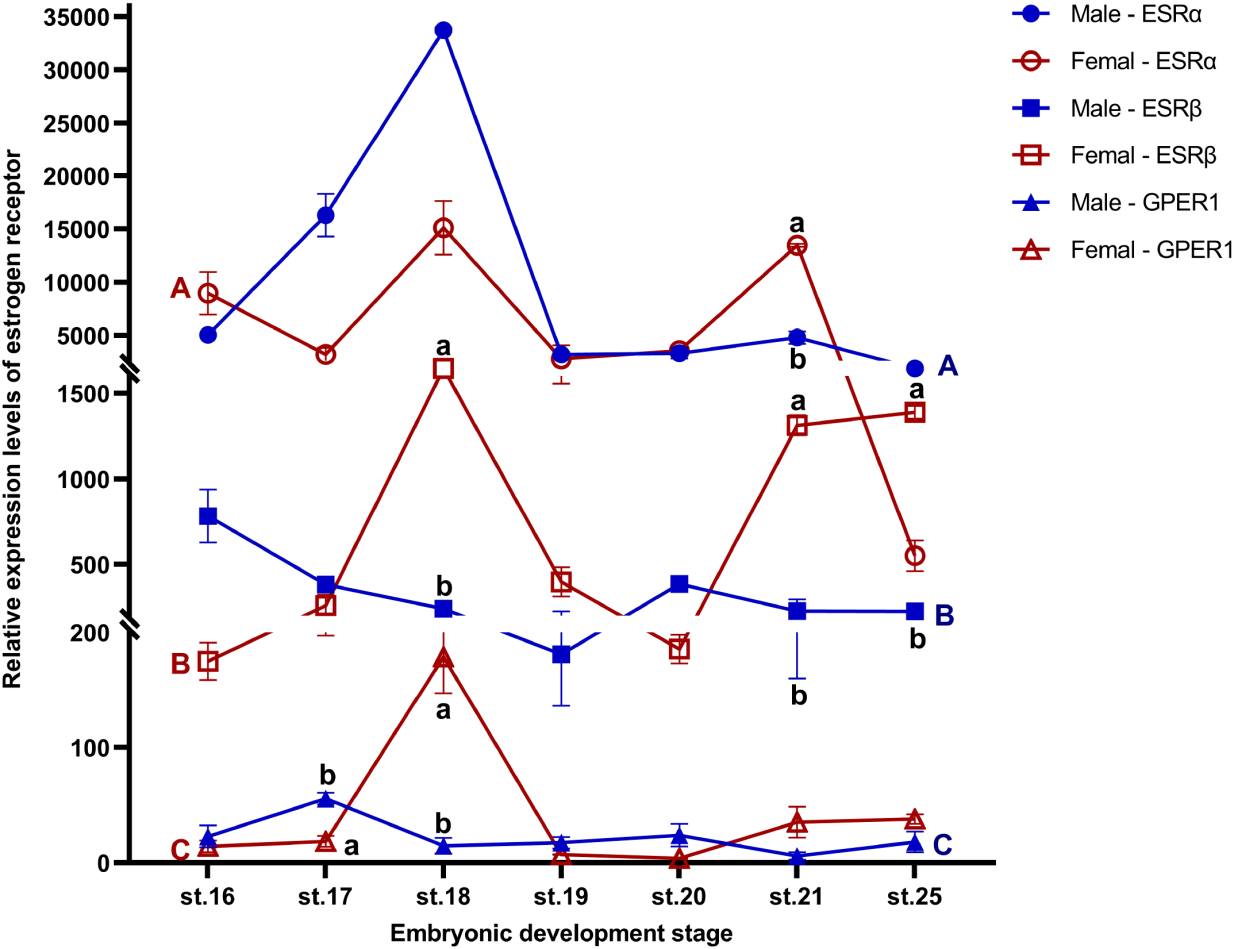
Expression profiles of *ESRα*, *ESRβ*, and *GPER1* during gonadal sex development (stages 16–25) in female and male *T. scripta*. Sex differences within the same developmental stage are indicated by different lowercase letters **(a, b)**. Developmental stage differences in males are marked with blue uppercase letters **(A-C)**, and developmental stage differences in females are marked with red uppercase letters **(A-C)**. No significant difference was found in gene expression at any developmental stage within each sex. Each experiment was independently repeated at least three times. All data are presented as means ± standard error of the mean (SEM). Significant effects were considered at *p* ≤ 0.05. Data are mean ± standard deviation (SD); *n* ≥ 3.

*ESRα*, *ESRβ*, and *GPER1* were constitutively expressed from stage 16, before the onset of gonadal differentiation in embryonic gonads of *T. scripta*, through to stage 25 of embryonic development at both FPT and MPT. The expression level of *GPER* at stage 21 in males was the relative point of relative expression. Specifically, ESRα expression was significantly higher in females than in males at stage 21; ESRβ expression was significantly higher in females than in males at stages 18, 21, and 25; GPER1 expression was significantly higher in males than in females at stage 17, but higher in females than in males at stage 18. There was no difference in expression levels of any receptor between MPT and FPT at other same stages (**Figure 1**; **Supplementary Table S3**). The results also showed that regardless of FPT or MPT, the expression levels of any of the three ESRs did not show significant changes during stage 16 to stage 21 and stage 25 (**Figure 1**; **Supplementary Tables S4–S6**). Furthermore, *ESRα* and *ESRβ* were robustly expressed in embryonic gonads, with *ESRα* mRNA level being higher than those of *ESRβ* in gonads of embryos. However, weaker expression of *GPER1* was detected in both FPT and MPT gonads compared to that of *ESRα* and *ESRβ* (**Figure 1**; **Supplementary Table S7**).

### 3.2 Estradiol treatments induced ovarian differentiation at MPT

**Figure 2:**
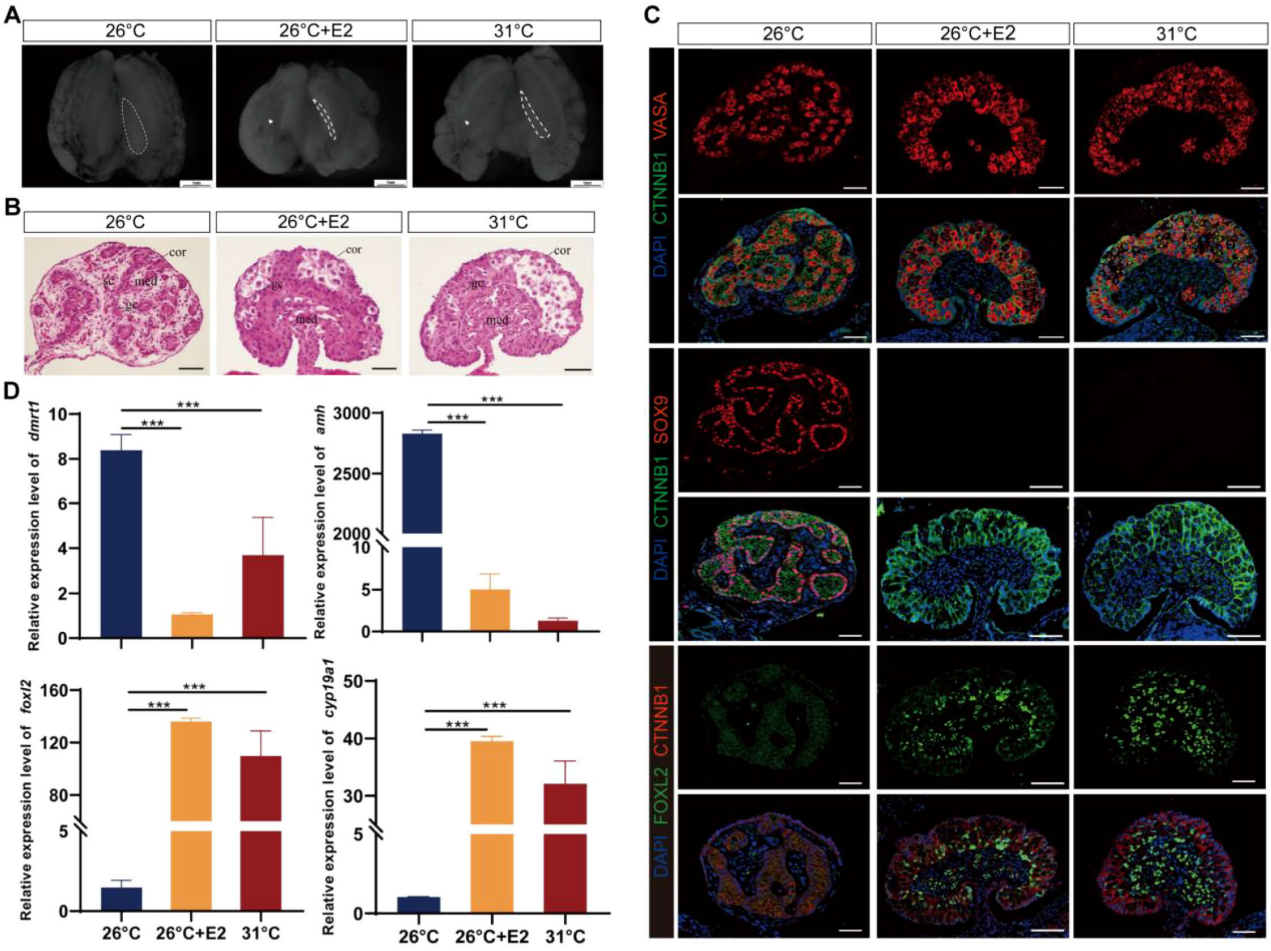
Estradiol treatments during temperature-sensitive period (TSP) at male-producing temperature (MPT)-induced ovarian differentiation in *T*. *scripta.* **(A)** Morphological analysis of the adrenal–kidney–gonad complex (AKG) in hatchings of control males, control females, and E_2_-induced females. The dashed black line indicates gonad. Gd, gland; Ovi, oviduct. Scale bars are 1 mm. **(B)** H&E-stained sections of AKG in hatchings of males, females, and E2-induced females. Cor, cortex region; Med: medullary region; Gc, germ cells; Sc, Sertoli cell. Scale bars are 50 μm. **(C)** Immunofluorescence images of VASA (red), CTNNB1, FOXL2 (green), SOX9 (red), and DAPI (4′,6-diamidino-2-phenylindole, blue) in gonadal cross sections of hatching males, females, and E_2_-induced females. **(D)** Relative expression of *Dmrt1*, *Amh*, *Foxl2*, and *Cyp19a1* mRNA in gonads of male, female, and E2-induced females. Three asterisks: *p* < 0.001.

Based on histological and immunofluorescence characteristics, embryos incubated at MPT displayed ovarian structure at stage 25 following E2 treatment (**Figures 2A–C**; **Supplementary Figure S1A**). Compared to the fetal gonads in MPT groups, which were short and round, gonads following estradiol treatments in MPT embryos became long and flat, resembling those in the FPT groups (**Figure 2A**). MPT control gonads exhibited well-differentiated testis cords with a degenerated cortex and dense medulla, and the primordial germ cells (PGCs) were arranged in the seminiferous cords, whereas FPT control gonads exhibited typical ovarian morphology, showing an advanced outer cortex compartment with PGCs (**Figure 2B**). For eggs treated with E2 at MPT, the morphology of gonads exhibited a completely male-to-female gonadal sex reversal phenotype (**Figure 2B**). MPT control gonads expressed SOX9, a marker of Sertoli cells, whereas FPT control gonads expressed FOXL2, a critical marker of granulosa cells (**Figure 2C**). In the gonads treated with E2 at MPT, FOXL2 expression showed a female-like pattern and SOX9 vanished (**Figure 2C**). In addition, compared with normal male controls, the expression of ovarian-related markers *Cyp19a1* and *Foxl2* significantly increased, whereas that of testis-related markers *Amh* and *Dmrt1* sharply decreased in gonads (stages 25) treated with E2 at MPT (**Figure 2D**).

### 3.3 Agonists of ESRα, ESRβ, or GPER1 induced ovarian differentiation at MPT

**Figure 3:**
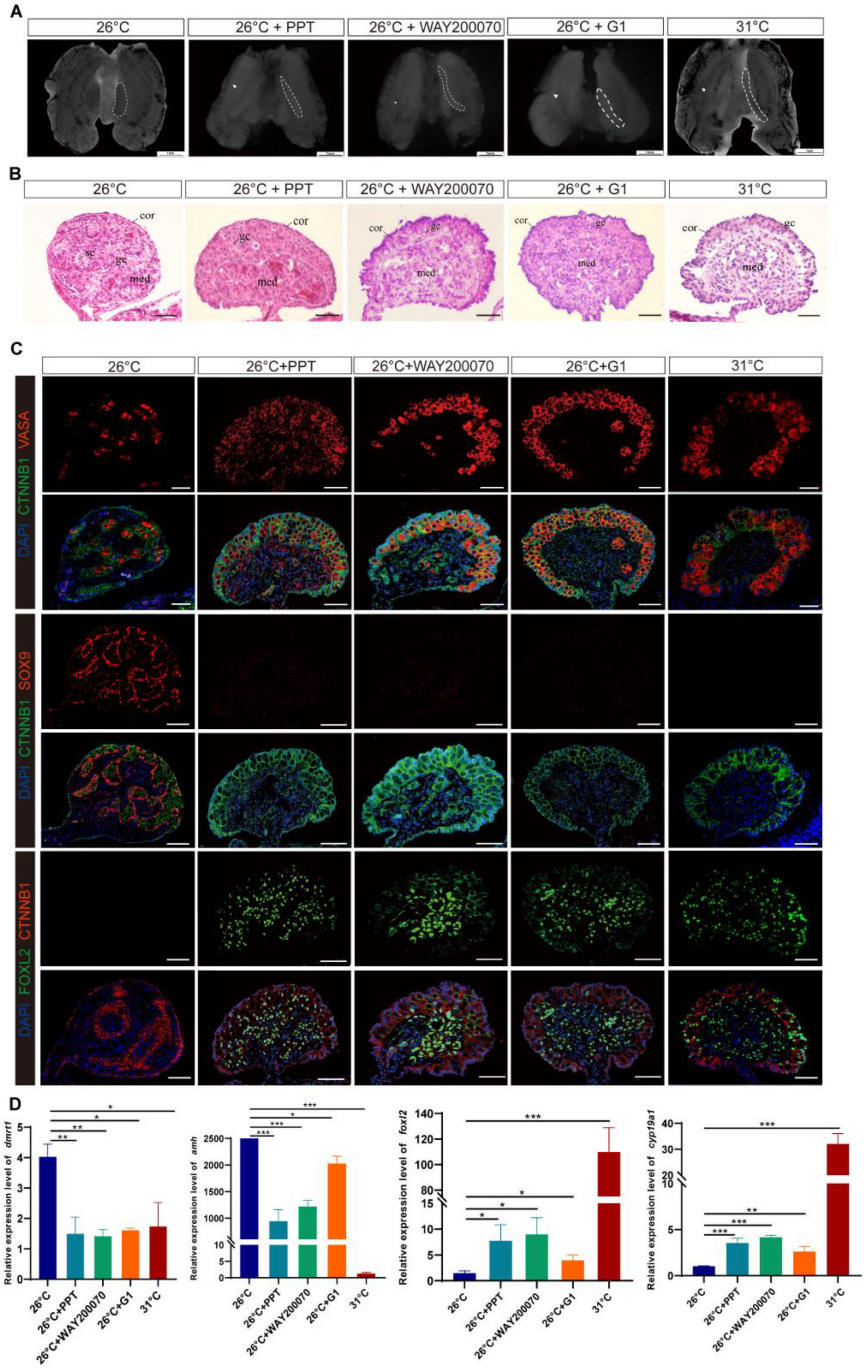
ESRα^+^-, ESRβ^+^-, or GPER1^+^-induced ovarian differentiation at male-producing temperature (MPT) in *T*. *scripta*. **(A)** Morphological analysis of the adrenal–kidney–gonad complex (AKG) in control male hatchings, control female hatchings, and hatchings incubated at 26°C treatment with ESRα^+^, ESRβ^+^, or GPER1^+^. The dashed black line indicates gonad. Gd, gland; Ovi, oviduct. Scale bars are 1 mm. **(B)** H&E-stained sections of AKG in control male hatchings, control female hatchings, and hatchings incubated at 26°C treatment with ESRα^+^, ESRβ^+^, or GPER1^+^. Cor, cortex region; Med: medullary region; Gc, germ cells; Sc, Sertoli cell. Scale bars are 50 μm. **(C)** Immunofluorescence images of VASA (red), CTNNB1, FOXL2 (green), SOX9 (red), and DAPI (4′,6-diamidino-2-phenylindole, blue) in gonadal cross sections from control male hatchings, control female hatchings, and hatchings incubated at 26°C treatment with ESRα^+^, ESRβ^+^, or GPER1^+^. **(D)** Relative expression of *Dmrt1*, *Amh*, *Foxl2*, and *Cyp19a1* mRNA in gonads of control male hatchings, control female hatchings, and hatchings incubated at 26°C treatment with ESRα^+^, ESRβ^+^, or GPER1^+^. One asterisk indicates statistical significance at *p* < 0.05; two asterisks: *p* < 0.01; three asterisks: *p* < 0.001.

Treatment with ESRα^+^, ESRβ^+^, and GPER1^+^ resulted in significantly more females than the control incubated at MPT (**Supplementary Figure S1B**, **Table 1**). Some embryos in the ESRα^+^, ESRβ^+^, and GPER1^+^ group exhibited ovarian differentiation with visible morphological and histological changes, showing typical long and flat ovaries with thickened outer cortex containing a number of germ cells and degenerated medullary cords as in FPT control (**Figures 3A, B**). Those feminized gonads expressed FOXL2 in granulosa cells of both the medulla and the cortex, similar to the FPT female type (**Figure 3C**). Gonadal tissues displayed a clear induction of sex-specific mRNA after embryo exposure to the specific agonists for the three ESRs. Treatment with PPT, WAY200070, and G1 alone increased the expression of ovarian-related markers *Cyp19a1* and *Foxl2* and decreased the expression of testis-related markers *Amh* and *Dmrt1* (**Figure 3D**). Furthermore, although these agonists increased the expression of *Cyp19a1* and *Foxl2* in the gonads compared to embryos incubated at 26°C, the expression levels did not exceed those observed in embryos incubated at 31°C.

**Table 1:** Effect of chemical drugs on sex determination at MPT.

Treatment	Number			Sex reversed ratio (%)	Chi-square	*p*-value
	Total	Male	Female			
E2	64	0	64	100.00	124.000	<0.001
ESRα^+^	44	34	10	22.73	15.087	<0.001
ESRβ^+^	52	42	10	19.23	12.670	<0.001
GPER1^+^	60	54	6	10.00	6.316	0.012
E2^+^Tripo^−^	64	32	32	50.00	40.435	<0.001

Embryos of the red-eared slider turtle (*T. scripta*) were incubated at male-producing temperature (MPT, 26°C) and treated with E2, ESRα^+^, ESRβ^+^, GPER1^+^, and a combination of estrogen and Tripo^−^. The chi-square test was employed to test the sex ratio with 95% confidence intervals between MPT and MPT treated with ESR agonists, as well as between MPT treated with E2 and MPT treated with a combination of E2 and three ESR antagonists.

### 3.4 Estrogen-induced feminization can be reversed by the combination of ESRα, ESRβ, and GPER1 antagonists

**Figure 4:**
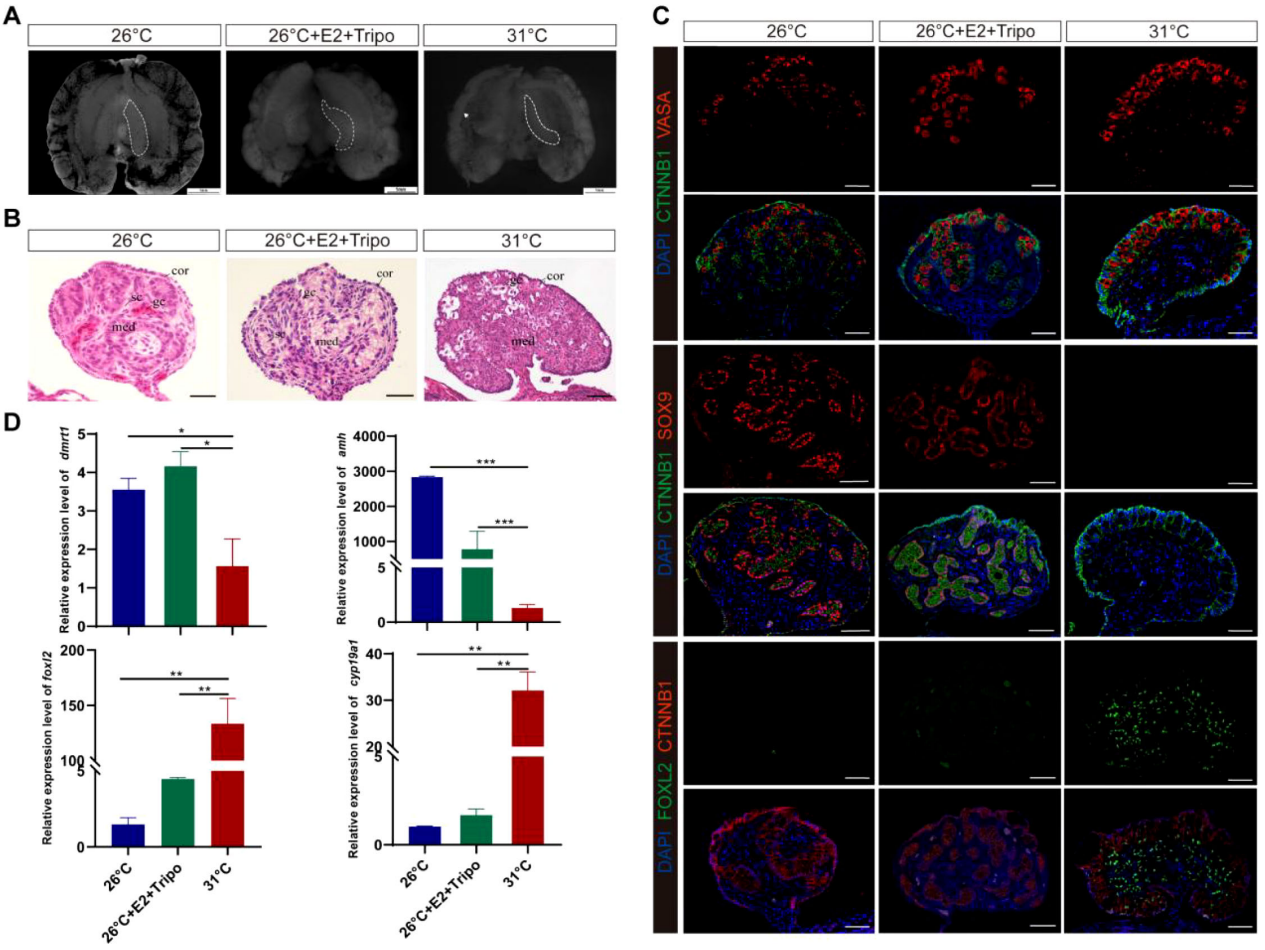
Estrogen-induced feminization can be reversed by the combination of ESRα^−^, ESRβ^−^, and GPER1^−^ in *T*. *scripta*. **(A)** Morphological analysis of the adrenal–kidney–gonad complex (AKG) in control male hatchings, control female hatchings, and hatchings incubated at 26°C treatment with E_2_, ESRα^−^, ESRβ^−^, and GPER1^−^. The dashed black line indicates gonad. Gd, gland; Ovi, oviduct. Scale bars are 1 mm. **(B)** H&E-stained sections of AKG in control male hatchings, control female hatchings, and hatchings incubated at 26°C treatment with E2, ESRα^−^, ESRβ^−^, and GPER1^−^. Cor, cortex region; Med: medullary region; Gc, germ cells; Sc, Sertoli cell. Scale bars are 50 μm. **(C)** Immunofluorescence images of VASA (red), CTNNB1, FOXL2 (green), SOX9 (red), and DAPI (4′,6-diamidino-2-phenylindole, blue) in gonadal cross sections from control male hatchings, control female hatchings, and hatchings incubated at 26°C treatment with E2, ESRα^−^, ESRβ^−^, and GPER1^−^. **(D)** Relative expression of *Dmrt1*, *Amh*, *Foxl2*, and *Cyp19a1* mRNA in gonads of control male hatchings, control female hatchings, and hatchings incubated at 26°C treatment with E2, ESRα^−^, ESRβ^−^, and GPER1^−^. One asterisk indicates statistical significance at *p* < 0.05; two asterisks: *p* < 0.01; three asterisks: *p* < 0.001.

E2-induced male-to-female sex reversal was rescued by the triple combination of ESRα^−^, ESRβ^−^, and GPER1^−^ (**Figure 4**; **Supplementary Figure S1C**), which exhibited a male-like phenotype with an attenuated outer cortex and an apparent medulla occupied by testicular-like cords in the E2 and Tripo− group (**Figures 4A, B**). Treatment with ESRα^−^, ESRβ^−^, and GPER1^−^ overrode E2-induced feminization of MPT embryos, which was characterized by the disappearance of the ovarian regulator FOXL2 in gonadal cells and ectopic expression of the male marker SOX9 in the nuclei of Sertoli cells in the seminiferous cords of gonads (**Figure 4C**). Testicular differentiation markers *Dmrt1* and *Amh* were strongly upregulated, and ovarian development regulators *Cyp19a1* and *Foxl2* were significantly downregulated in E2 and Tripo− gonads, which are similar to the sex-specific expression pattern of gonads in MPT rather than FPT (**Figure 4D**). In addition, combined treatment with E2 and three ESR antagonists resulted in the development of some individuals with ovotestes, which expressed both SOX9 and FOXL2 (**Supplementary Figure S2**). This suggests that blocking ESR activity partially disrupts ovarian development, leading to an intermediate gonadal state.

### 3.5 Antagonists of ESRα, ESRβ, or/and GPER1 do not disrupt ovarian differentiation at FPT

However, eggs treated with ESRα^−^, ESRβ^−^, or/and GPER1^−^ showed no significant difference in their gonadal sex ratio compared with the FPT control. Gonads in the FPT + AZD9496, FPT + PHTPP, and FPT + G15 groups displayed topical long and flat ovaries with advanced outer cortex and germ cells like those in the FPT control group (**Supplementary Figures S3A, B**). FOXL2 expression was identified in granulosa cells of both the medulla and the cortex, and no male marker SOX9 was detected in the gonadal cells of eggs that received AZD9496, PHTPP, or G15 treatment (**Supplementary Figure S3C**). Transcripts of the ovarian development regulator *Cyp19a1* and *Foxl2* increased while transcripts of testicular differentiation markers *Amh* and *Dmrt1* remained at low levels in gonads when eggs were treated with AZD9496, PHTPP, or G15, which are consistent with those incubated at FPT (**Supplementary Figure S3D**). Then, we added three antagonists of ESRs together to turtle eggs to assess whether ESRα, ESRβ, and GPER1 have a compensatory function for each other, and no apparent morphological alteration in the gonads of the FPT + Tripo group (**Supplementary Figures S4A, B**). Immunofluorescent detection revealed that FOXL2 was robustly expressed in somatic cells of gonads and SOX9 was not detected in gonads of the FPT + Tripo group, similar to the control group incubated at FPT (**Supplementary Figure S4C**). RT-PCR analysis showed that *Amh*, *Dmrt1*, *Cyp19a1*, and *Foxl2* transcripts in gonads after treatment with AZD9496, PHTPP, and G15 together exhibited the same expression pattern as that incubated at FPT (**Supplementary Figure S4D**).

## 4 Discussion

The mechanism by which temperature directs the expression of genes and cell signaling to induce the differentiation of the bipotential gonad to the testis or ovary is the most intriguing question in TSD species. However, estrogens have been shown to be necessary and sufficient for ovarian determination and differentiation in TSD reptiles (7, 10, 19, 23, 49, 50). Our study provides insights into the mechanisms through which estrogens influence embryo sex at MPT: estrogens exert their effects through three ESRs (ESRα, ESRβ, and GPER1), thereby regulating the expression of estrogen-responsive sex-related genes to induce ovarian differentiation and inhibit testis differentiation at MPT, whereas at FPT, the expression of female-specific regulators responsible for ovarian development is directly regulated by warmer temperatures as a primary cue and does not require support from ESRs. This finding not only improves our understanding of the involvement of estrogen in sex determination in TSD species, but also carries broader implications for estrogen-associated gonadal development in vertebrates.

ESRs are expected to mediate estrogen’s effects on embryo sex in vertebrates, but the engagement of ESRs varies from species to species. In teleost, there are three nuclear ESRs, ESRα, ESRβ1, and ESRβ2, encoded by genes, *Esr1*, *Esr2a*, and *Esr2b*, and ESRβ1 and ESRβ2 are required to mediate the role of estrogen in sex determination (51). In contrast, the feminization effect of estrogen on sexual differentiation is mediated by ESRα in birds and turtles with GSD (genetic sex determination) (31, 52). For example, the selective ESRα agonist induced ovarian differentiation of genetic male chicken (*Gallus gallus*), Japanese quail (*Coturnix japonica*), and Chinese soft-shelled turtle (*Pelodiscus sinensis*), but the selective ESRβ agonist did not (31, 37, 52, 53). The only study on the functionalization of ESRs in TSD reptiles demonstrated that ESRα, rather than ESRβ, modulates estrogen-induced sex reversal in ovarian differentiation in American alligator (*Alligator mississippiensis*) (30, 36). However, our study showed that application of a specific agonist of ESRα, ESRβ, or GPER1 can induce feminization at MPT, and the male-to-female sex reversal induced by E2 can be reversed by the combination of ESRα, ESRβ, and GPER1 antagonists in red-eared sliders. These results indicated that estrogen induces ovarian differentiation through the three ESRs (ESRα, ESRβ, and GPER1) in *T*. *scripta* at MPT. This interspecies variation in the involvement of ESRs may be related to different TSD patterns, as the MPT of the American alligator with a TSD II pattern significantly differs from that of the red-eared slider turtle with a TSD Ia pattern (19, 45), and temperature can affect conformational transitions of ESRs (54, 55). Additionally, the temporal and spatial expression patterns of ESRs may shed light on their respective physiological functions (17, 56). We found that ESRα, ESRβ, and GPER1 in gonads express at stage 16, which is early in TSP and clearly prior to gonadal differentiation, at both MPT and FPT in *T*. *scripta*. The mRNA expression levels of ESRs within each stage exhibited the pattern of *ESRα* > *ESRβ* > *GPER1* (**Figure 1**; **Supplementary Table S7**), which is consistent with the pattern of sex reversal ratio by the administration of agonists of ESRα, ESRβ, and GPER1. Therefore, ESRα, ESRβ, and GPER1 mediate estrogen action on sex determination and ovarian differentiation in *T*. *scripta* at MPT. Nonetheless, because our knowledge is limited to two species, further studies are urgently needed to comprehensively understand the role of ESRs in sex determination in TSD reptiles.

It is known that these two sexual pathways are mutually antagonistic, and activating one of the alternatives and repressing the other is critical for the maintenance of the testis or ovary programs (3, 57). Our study demonstrated that agonists of ESRs (ESRα, ESRβ, or GPER1) increased the expression of the ovarian regulators *Foxl2* and *Cyp19a1* (6, 58–63) and decreased the expression of the testicular Sertoli cell markers *Dmrt1* and *Amh* (25, 59, 64, 65). Agonists of ESRs also activated the ovarian regulator of FOXL2 (58–60) and repressed an indispensable regulator of SOX9 (24, 26) for testis differentiation in *T*. *scripta*. Furthermore, treatment with ESRα, ESRβ, and GPER1 antagonists together reverses the estrogen-induced feminization of *T. scripta*, with *Dmrt1*, *Sox9*, and *Amh* increasing and *FoxL2* and *Cyp19a1* decreasing (**Figure 4**), confirming that ESRα, ESRβ, and GPER1 mediate the estrogen-induced feminization of *T. scripta* incubated at MPT. Estrogen–ESR signaling diverts the bipotential gonad of embryos to females by strongly altering the expression patterns of sex-related genes (24, 66, 67). For example, the direct effects of sex steroids on *Amh* transcription are mediated by ESRα action on *Amh* promoter sequences, and modest estrogen action is also mediated by the membrane G-coupled estrogen receptor 1 (68). *Foxl2* binds directly to ESRβ, regulating estrogen production in granulosa cells in mice, and this transcription factor, together with ESRα, synergizes repression of *Sox9* by negatively regulating the testis-specific enhancer core element unit of the promoter (69, 70). Although further experiments are needed to demonstrate a direct relationship between ESRs and these sex-related genes in *T*. *scripta*, these results collectively point towards estrogen signaling, with ESRα, ESRβ, and GPER1 regulating the expression of estrogen-responsive sex-related genes, inducing ovarian differentiation, and inhibiting testis differentiation of *T*. *scripta* at MPT.

Our results showed that treatment with any of the three antagonists individually or all three antagonists combined did not affect ovarian development in *T. scripta* (**Supplementary Figures S3, S4**). This confirms that ESRs are not required for regulating female-specific gene expression underlying ovarian development at FPT. Together with previous findings that warmer temperatures influence the expression of sex-related genes involved in ovarian determination and differentiation in *T. scripta* (6, 10), our results suggest that warmer temperatures alone can drive the feminization process, independent of ESR signaling. For example, warmer temperature can repress expression of an essential regulator of *Dmrt1* for testicular differentiation and promote expression of an important regulator *Foxl2* for ovarian differentiation in *T*. *scripta* at FPT (6, 10, 64). Specifically, at warmer temperatures (FPT), signal transducer and activator of transcription 3 (STAT3) is phosphorylated, binds the *Kdm6b* locus, and represses *Dmrt1* expression, blocking the male pathway; meanwhile, pSTAT3 also binds the *Foxl2* locus and promotes *Foxl2* expression, activating the female pathway (6, 10, 64). Recent studies showed that embryos incubated at higher temperatures have more germ cells (GCs) than those incubated at the male-inducing temperature. Furthermore, elimination of GCs in embryos incubating at intermediate temperatures results in a strong shift toward male-biased sex ratios. These findings suggested that warmer temperatures directly increase germ cell number and a higher number of GCs favor the female pathway in *T*. *scripta* (71, 72). Although exogenous estrogen at MPT appears to be the physiological equivalent of warmer incubation temperature, both result in ovarian development (7, 17, 73), and exogenous estrogen produces the gonads whose morphology and histology differ from those induced by temperature (24, 66, 74–77). For example, in *T*. *scripta*, ovaries that develop in embryos incubating at FPT are large and thick, whereas ovaries in embryos incubating at MPT treated with exogenous E2 are small in size and the oviducts are not fully detached from the underlying mesonephric tissues (56, 66, 78). Those studies combined with our results indicated different molecular regulatory mechanisms between warmer temperature- and estrogen-induced feminization for *T*. *scripta*.

More generally, genetic effects are essential for sex differentiation in mammals and birds with heteromorphic sex chromosomes, whereas estrogen is a significant player involved in sex differentiation in ectothermic vertebrates, in which sex chromosomes are rarely differentiated. In TSD reptiles, both temperature and estrogens can trigger ovarian development through differential activation/repression of sex-determining genes during the temperature-sensitive period. The molecular mechanism of how estrogen promotes ovarian differentiation through ovary regulatory loops is critically important to understanding gonad differentiation. Estrogen regulates the expression of estrogen-responsive sex-related genes through the three ESRs—ESRα, ESRβ, and/or GPER1—to induce ovarian differentiation of *T*. *scripta* at MPT. Clearly, this finding broadens our knowledge of the role of ESRs in sex determination and ovarian differentiation.

## Data Availability

The original contributions presented in the study are included in the article/**Supplementary Material**. Further inquiries can be directed to the corresponding author.
