# Supplementary material for: Estrogen receptor and temperature independently influence sex determination in the red-eared slider turtle
Source: Front Endocrinol (Lausanne). 2025 Jul 21;16:1632672. doi: 10.3389/fendo.2025.1632672 (PMC12318736; doi:10.3389/fendo.2025.1632672)
Supplement: Supplementary file 1 [file DataSheet1.docx]

**Estrogen receptor and temperature independently**

**influence sex determination in the red-eared slider turtle**

**Supplementary materials**


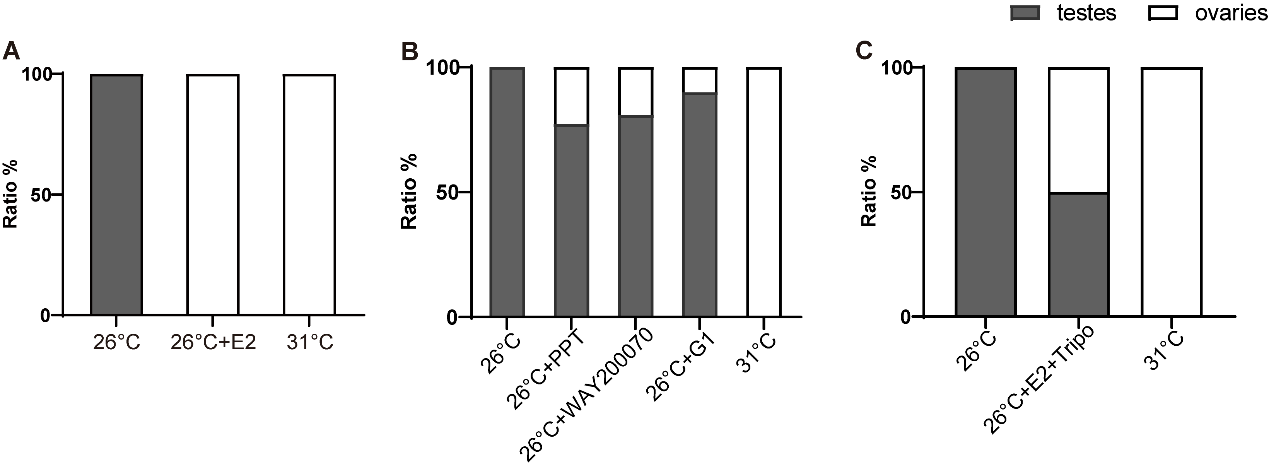
**Fig. S1 The number of phenotypic sex in each treatment group at MPT in *T*. *scripta*.** (A) The numbers of phenotypical sex of embryo incubated at 26°C, 31°C and embryo incubated at 26°C treatment with E2. (B) The numbers of phenotypical sex of embryo incubated at 26°C in response to PPT, WAY200070, and G1 treatments. (C) The numbers of phenotypical sex of embryo incubated at 26°C in response to combination effect of E2, AZD9496, PHTPP and G15.

**
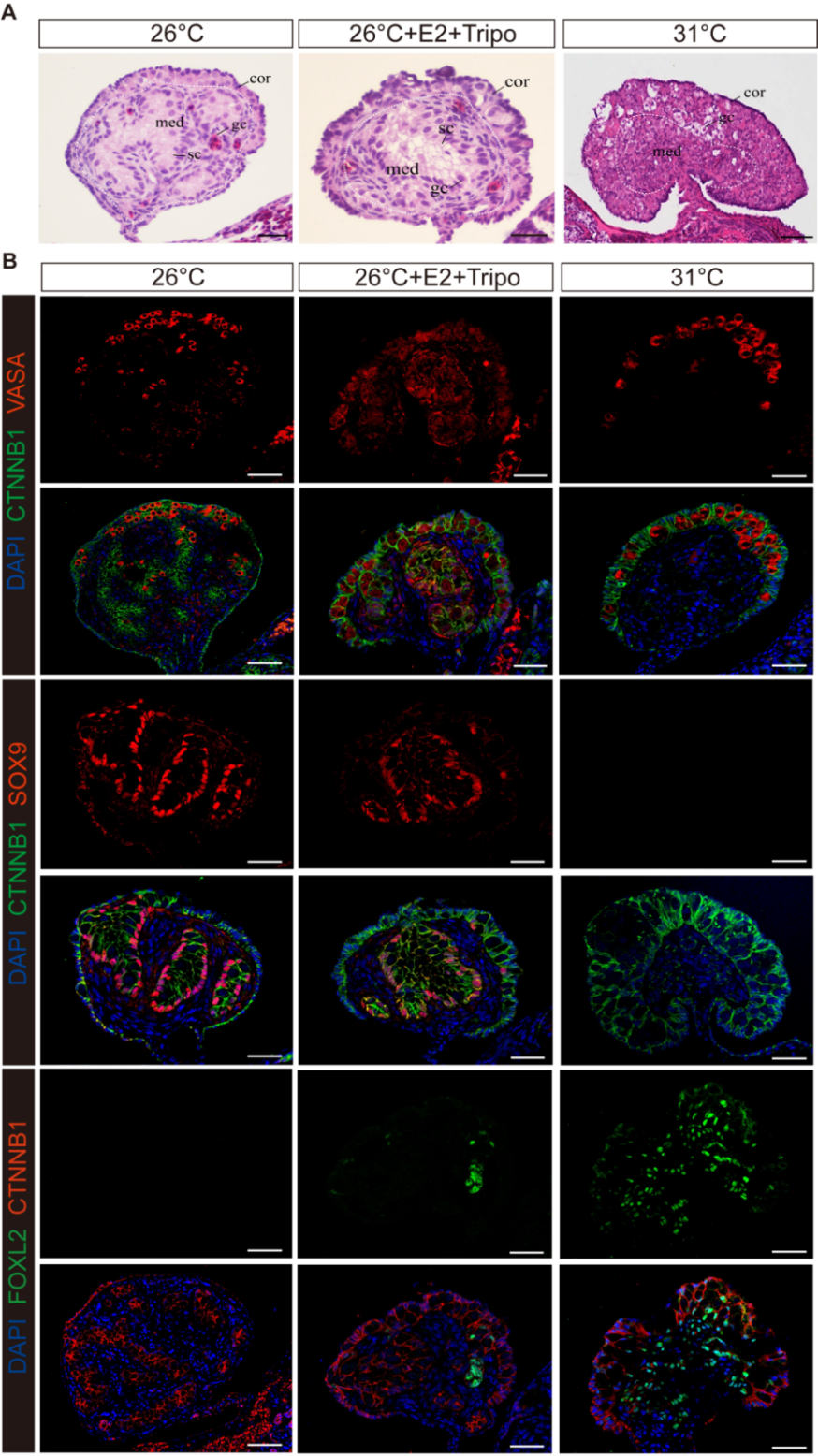
**

**Fig. S2 Intersex emerged from treatment with a combination antagonists of estrogen , ESRα (AZD9496), ESRβ (PHTPP), and GPER1 (G15).** (A) H&E stain the cross sections of AKG of control male hatchings, control female hatchings and hatchings which incubated at 26°C treatment with E_2_, AZD9496, PHTPP and G15. Cor, cortex region; Med: medullary region; Gc, germ cells; Sc, Sertoli cell. Scale bars is 50 μm. (B) Immunoﬂuorescence images of VASA (red), CTNNB1, FOXL2 (green), SOX9 (red) and DAPI (4′,6-diamidino-2-phenylindole, blue) in gonadal cross sections from control male hatchings, control female hatchings and hatchings which incubated at 26°C treatment with E2, AZD9496, PHTPP and G15.


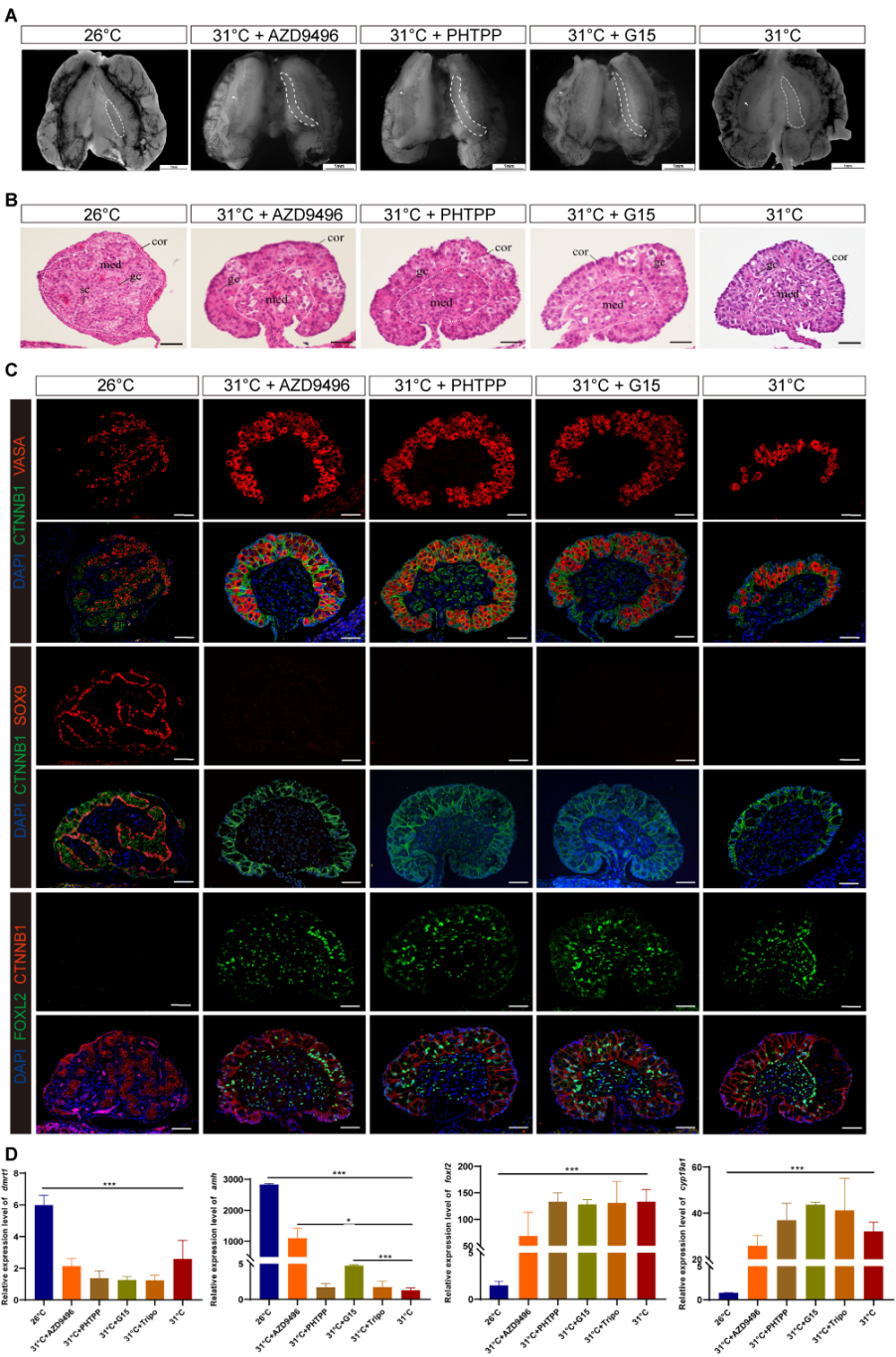


**Fig. S3** **Antagonists of ESRα (AZD9496), ESRβ (PHTPP) or GPER1 (G15) do not disrupt ovarian differentiation at female producing temperature (FPT) in *T*. *scripta*.** (A) Morphological analysis of the adrenal-kidney-gonad complex (AKG) in control male hatchings, control female hatchings and hatchings incubated at 31 °C treatment with AZD9496, PHTPP or G15. The dashed black line indicates gonad. Gd, gland; Ovi, oviduct. Scale bars are 1 mm. (B) H&E stained sections of AKG in control male hatchings, control female hatchings and hatchings incubated at 31°C treatment with AZD9496, PHTPP or G15. Cor, cortex region; Med: medullary region; Gc, germ cells; Sc, Sertoli cell. Scale bars are 50 μm. (C) Immunoﬂuorescence images of VASA (red), CTNNB1, FOXL2 (green), SOX9 (red) and DAPI (4′,6-diamidino-2-phenylindole, blue) in gonadal cross sections from control male hatchings, control female hatchings and hatchings incubated at 31 °C treatment with AZD9496, PHTPP or G15. (D) Relative expression of *Dmrt1*, *Amh*, *Foxl2* and *Cyp19a1* mRNA in gonads of control male hatchings, control female hatchings and hatchings incubated at 31 °C treatment with AZD9496, PHTPP or G15. Two asterisks: P < 0.01; three asterisks: P < 0.001.


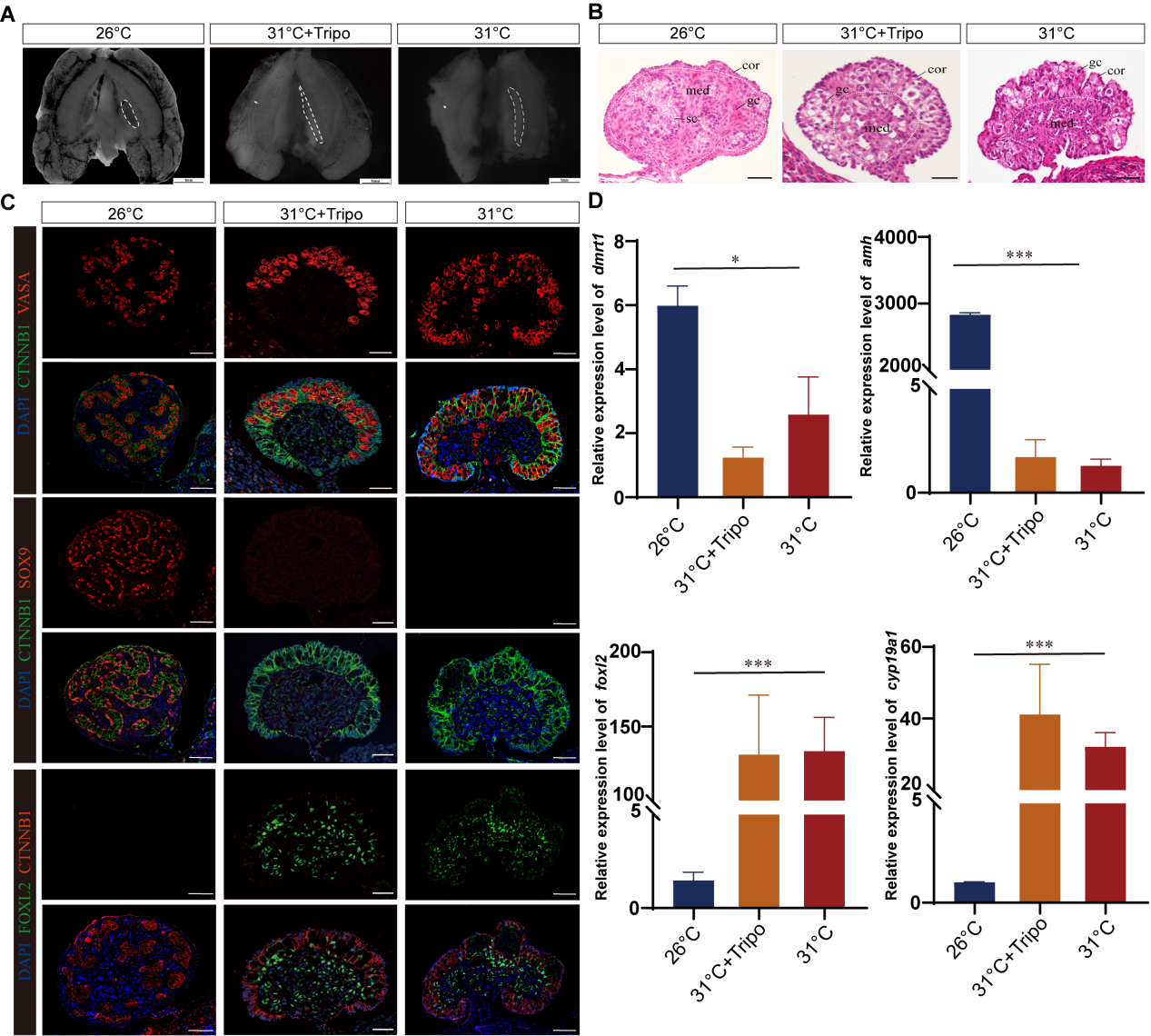


**Fig. S4 Triple combination of antagonists of ESRα, ESRβ and GPER1 does not significantly disrupt ovarian differentiation at FPT in *T*. *scripta*.** (A) Morphology analysis of the adrenal-kidney-gonad complex (AKG) in control male hatchings, control female hatchings and hatchings which incubated at 31°C treatment with AZD9496, PHTPP and G15. The dashed black line indicates gonad. Gd, gland; Ovi, oviduct. Scale bars is 1 mm. (B) H&E stain the cross sections of AKG of control male hatchings, control female hatchings and hatchings which incubated at 31°C treatment with AZD9496, PHTPP and G15. Cor, cortex region; Med: medullary region; Gc, germ cells; Sc, Sertoli cell. Scale bars is 50 μm. (C) Immunoﬂuorescence images of VASA (red), CTNNB1, FOXL2 (green), SOX9 (red) and DAPI (4′,6-diamidino-2-phenylindole, blue) in gonadal cross sections from control male hatchings, control female hatchings and hatchings which incubated at 31°C treatment with AZD9496, PHTPP and G15. (D) Relative expression of *Dmrt1*, *Amh*, *Foxl2* and *Cyp19a1* mRNA in gonads of control male hatchings, control female hatchings and hatchings which incubated at 31°C treatment with AZD9496, PHTPP and G15.

**Table S1 Embryos with chemical treatment**

| Treatment group | Chemical | Chemical concentration | Incubation temperature |
| --- | --- | --- | --- |
| MPT+E2 | E2 | 0.5 μg/g 17β-estradiol (E2) | 26 °C |
| MPT+PPT | ESRα+ | 5 μg/g ESRα agonist (PPT) | 26 °C |
| MPT+ WAY200070 | ESRβ+ | 5 μg/g ESRβ agonist (WAY200070) | 26 °C |
| MPT+G1 | GPER1+ | 0.25 μg/g GPER1 agonist (G1) | 26 °C |
| MPT+E2+Tripo | E2 and Tripo- | 0.5 μg/g 17β-estradiol (E2), antagonists of 5 μg/g ESRα (AZD9496), 5 μg/g ESRβ (PHTPP) and 0.25 μg/g GPER1 (G15) | 26 °C |
| FPT+AZD9496 | ESRα- | 5 μg/g ESRα antagonist (AZD9496) | 32 °C |
| FPT+PHTPP | ESRβ- | 5 μg/g ESRβ antagonist (PHTPP) | 32 °C |
| FPT+G15 | GPER1- | 0.25 μg/g GPER1 antagonist (G15) | 32 °C |
| FPT+Tripo | Tripo- | Antagonists of 5 μg/g ESRα (AZD9496), 5 μg/g ESRβ (PHTPP), and 0.25 μg/g GPER1 (G15) | 32 °C |

Embryos of the red-eared slider turtle (*T. scripta*) groups treatment as following : MPT + E2 (eggs with 0.5 μg/g E2 at MPT), MPT + PPT (eggs with 5 μg/g PPT at MPT), MPT + WAY200070 (eggs with 5 μg/g WAY200070 at MPT), MPT + G1 (eggs with 0.25 μg/g G1 at MPT), FPT + AZD9496 (eggs with 5 μg/g AZD9496 at FPT), FPT + PHTPP (eggs with 5 μg/g PHTPP at FPT), FPT + G15 (eggs with 0.25 μg/g at FPT), MPT + E2 + Tripo (eggs with 0.5 μg/g E2, 5 μg/g AZD9496, 5 μg/g PHTPP and 0.25 μg G15 at MPT), and FPT + Tripo (5 μg/g AZD9496, 5 μg/g PHTPP and 0.25 μg G15 at FPT). The dose of chemical are referred to the previous studies on reptiles (Doheny et al., 2016; Kohno et al., 2015; Li et al., 2022; Toyota et al., 2020).

**Table S2 Primer list for qRT-PCR**

| genes | Primer sequences (5’-3’) |
| --- | --- |
| *Dmrt1-*F | ACTACCCTCCTGCCTCCTACCT |
| *Dmrt1-*R | CTCCTTTGGTGCTTTCATTGCT |
| *Foxl2-*F | ACGAGTGCTTCATCAAGGTGCCC |
| *Foxl2-*R | TCCGCCGCCGTCGGTAGTT |
| *Gapdh-*F | TCTCTGTCGTGGACCTGACT |
| *Gapdh-*R | ACAGGAGACAACCTGGTCCT |
| *AMH-*F | CGGCTACTCCTCCCACACG |
| *AMH-*R | CCTGGCTGGAGTATTTGACGG |
| *Cyp19a1-*F | AATGATGAAGGCAATCCTGGT |
| *Cyp19a1-*R | GGCTGTCTTTCCTTCGGGTG |
| *ESRα*-F | GCTGCAGTCAGTCTTCTCTGA |
| *ESRα*-R | TTGGGCTTGATGTCTGCCTT |
| *ESRβ*-F | TCCTAATGTGTTGCTGGTGAGTCG |
| *ESRβ*-R | GGCCCAACCAATCATGTGAACCAA |
| *GPER1*-F | ATAAAAGCACACAAACACAGGAGT |
| *GPER1*-R | CAGGTAGCCAGCAGATAAAGAAAA |

**Table S3** **Differences** **in gene expression of each estrogen receptor at different incubation temperatures during the same stage**

| genes | stage | t | df | *p* |
| --- | --- | --- | --- | --- |
| *ESRα* | st.16 | -1.518 | 2 | 0.268 |
| *ESRα* | st.17 | 5.082 | 2 | **0.037** |
| *ESRα* | st.18 | 5.980 | 2 | **0.027** |
| *ESRα* | st.19 | 0.262 | 2 | 0.818 |
| *ESRα* | st.20 | 1.420 | 2 | 0.291 |
| *ESRα* | st.21 | -11.551 | 2 | **0.007** |
| *ESRα* | st.25 | 6.074 | 2 | **0.026** |
| *ESRβ* | st.16 | 3.187 | 2 | 0.086 |
| *ESRβ* | st.17 | 1.500 | 3 | 0.231 |
| *ESRβ* | st.18 | -26.847 | 2 | **0.001** |
| *ESRβ* | st.19 | -1.844 | 2 | 0.206 |
| *ESRβ* | st.20 | 7.616 | 3 | **0.005** |
| *ESRβ* | st.21 | -9.860 | 2 | **0.010** |
| *ESRβ* | st.25 | -22.544 | 3 | **0.000** |
| *GPER1* | st.16 | 0.660 | 2 | 0.577 |
| *GPER1* | st.17 | 5.387 | 4 | **0.006** |
| *GPER1* | st.18 | -5.057 | 4 | **0.007** |
| *GPER1* | st.19 | 1.615 | 3 | 0.205 |
| *GPER1* | st.20 | 2.058 | 4 | 0.109 |
| *GPER1* | st.21 | -2.113 | 4 | 0.102 |
| *GPER1* | st.25 | -1.633 | 3 | 0.201 |

**Table S4** **Differences in gene expression at each developmental stage of *ESRα* under MPT or FPT**

| genes | comparison | 26℃/MPT | | | | | 31℃/FPT | | | | |
| --- | --- | --- | --- | --- | --- | --- | --- | --- | --- | --- | --- |
|  |  | kruskal_statistic | kruskal_p_value | dunn_z_value | dunn_p_value | *dunn_p_adjusted* | kruskal_statistic | kruskal_p_value | dunn_z_value | dunn_p_value | *dunn_p_adjusted* |
| *ESRα* |  | -1.518 | 1.232 |  |  |  | 11.657 | 0.0701 |  |  |  |
|  | st.16- st.17 |  |  | 0.598 | 0.55 | 0.664 |  |  | -0.956 | 0.339 | 0.475 |
|  | st.16- st.18 |  |  | 1.076 | 0.282 | 0.494 |  |  | 0.717 | 0.473 | 0.585 |
|  | st.16- st.19 |  |  | -1.315 | 0.189 | 0.396 |  |  | -0.956 | 0.339 | 0.475 |
|  | st.16- st.20 |  |  | -0.837 | 0.403 | 0.564 |  |  | -0.956 | 0.339 | 0.475 |
|  | st.16- st.21 |  |  | -0.239 | 0.811 | 0.811 |  |  | 0.717 | 0.473 | 0.585 |
|  | st.16- st.25 |  |  | -1.793 | 0.073 | 0.255 |  |  | -1.912 | 0.056 | 0.220 |
|  | st.17- st.18 |  |  | 0.478 | 0.633 | 0.664 |  |  | 1.673 | 0.094 | 0.220 |
|  | st.17- st.19 |  |  | -1.912 | 0.056 | 0.234 |  |  | 0 | 1 | 1 |
|  | st.17- st.20 |  |  | -1.434 | 0.151 | 0.396 |  |  | 0 | 1 | 1 |
|  | st.17- st.21 |  |  | -0.837 | 0.403 | 0.564 |  |  | 1.673 | 0.094 | 0.22 |
|  | st.17- st.25 |  |  | -2.39 | 0.017 | 0.118 |  |  | -0.956 | 0.339 | 0.475 |
|  | st.18- st.19 |  |  | -2.39 | 0.017 | 0.118 |  |  | -1.673 | 0.094 | 0.220 |
|  | st.18- st.20 |  |  | -1.912 | 0.056 | 0.234 |  |  | -1.673 | 0.094 | 0.220 |
|  | st.18- st.21 |  |  | -1.315 | 0.189 | 0.396 |  |  | 0 | 1 | 1 |
|  | st.18- st.25 |  |  | -2.869 | 0.004 | 0.087 |  |  | -2.63 | 0.009 | 0.09 |
|  | st.19- st.20 |  |  | 0.478 | 0.633 | 0.664 |  |  | 0 | 1 | 1 |
|  | st.19- st.21 |  |  | 1.076 | 0.282 | 0.494 |  |  | 1.673 | 0.094 | 0.220 |
|  | st.19- st.25 |  |  | -0.478 | 0.633 | 0.664 |  |  | -0.956 | 0.339 | 0.475 |
|  | st.20- st.21 |  |  | 0.598 | 0.55 | 0.664 |  |  | 1.673 | 0.094 | 0.220 |
|  | st.20- st.25 |  |  | -0.956 | 0.339 | 0.548 |  |  | -0.956 | 0.339 | 0.475 |
|  | st.21- st.25 |  |  | -1.554 | 0.12 | 0.361 |  |  | -2.630 | 0.009 | 0.090 |

| genes | comparison | 26℃/MPT | | | | | 31℃/FPT | | | | |
| --- | --- | --- | --- | --- | --- | --- | --- | --- | --- | --- | --- |
|  |  | kruskal_statistic | kruskal_p_value | dunn_z_value | dunn_p_value | *dunn_p_adjusted* | kruskal_statistic | kruskal_p_value | dunn_z_value | dunn_p_value | *dunn_p_adjusted* |
| *ESRβ* |  | 10.629 | 0.101 |  |  |  | 13.840 | 0.0315 |  |  |  |
|  | st.16- st.17 |  |  | -0.717 | 0.473 | 0.710 |  |  | 0.470 | 0.638 | 0.744 |
|  | st.16- st.18 |  |  | -2.032 | 0.042 | 0.221 |  |  | 2.576 | 0.01 | 0.105 |
|  | st.16- st.19 |  |  | -2.510 | 0.012 | 0.221 |  |  | 1.090 | 0.276 | 0.527 |
|  | st.16- st.20 |  |  | -0.717 | 0.473 | 0.71 |  |  | 0.181 | 0.856 | 0.856 |
|  | st.16- st.21 |  |  | -2.032 | 0.042 | 0.221 |  |  | 1.783 | 0.075 | 0.200 |
|  | st.16- st.25 |  |  | -2.032 | 0.042 | 0.221 |  |  | 2.134 | 0.033 | 0.138 |
|  | st.17- st.18 |  |  | -1.315 | 0.189 | 0.33 |  |  | 2.352 | 0.019 | 0.131 |
|  | st.17- st.19 |  |  | -1.793 | 0.073 | 0.255 |  |  | 0.724 | 0.469 | 0.608 |
|  | st.17- st.20 |  |  | 0 | 1 | 1 |  |  | -0.324 | 0.746 | 0.825 |
|  | st.17- st.21 |  |  | -1.315 | 0.189 | 0.33 |  |  | 1.483 | 0.138 | 0.29 |
|  | st.17- st.25 |  |  | -1.315 | 0.189 | 0.33 |  |  | 1.861 | 0.063 | 0.200 |
|  | st.18- st.19 |  |  | -0.478 | 0.633 | 0.781 |  |  | -1.486 | 0.137 | 0.290 |
|  | st.18- st.20 |  |  | 1.315 | 0.189 | 0.33 |  |  | -2.641 | 0.008 | 0.105 |
|  | st.18- st.21 |  |  | 0 | 1 | 1 |  |  | -0.793 | 0.428 | 0.608 |
|  | st.18- st.25 |  |  | 0 | 1 | 1 |  |  | -0.687 | 0.492 | 0.608 |
|  | st.19- st.20 |  |  | 1.793 | 0.073 | 0.255 |  |  | -1.013 | 0.311 | 0.544 |
|  | st.19- st.21 |  |  | 0.478 | 0.633 | 0.781 |  |  | 0.694 | 0.488 | 0.608 |
|  | st.19- st.25 |  |  | 0.478 | 0.633 | 0.781 |  |  | 0.941 | 0.347 | 0.56 |
|  | st.20- st.21 |  |  | -1.315 | 0.189 | 0.33 |  |  | 1.773 | 0.076 | 0.200 |
|  | st.20- st.25 |  |  | -1.315 | 0.189 | 0.33 |  |  | 2.184 | 0.029 | 0.138 |
|  | st.21- st.25 |  |  | 0 | 1 | 1 |  |  | 0.181 | 0.856 | 0.856 |

**Table S5 Differences in gene expression at each developmental stage of *ESRβ* under MPT or FPT**

| genes | comparison | 26℃/MPT | | | | | 31℃/FPT | | | | |
| --- | --- | --- | --- | --- | --- | --- | --- | --- | --- | --- | --- |
|  |  | kruskal_statistic | kruskal_p_value | dunn_z_value | dunn_p_value | *dunn_p_adjusted* | kruskal_statistic | kruskal_p_value | dunn_z_value | dunn_p_value | *dunn_p_adjusted* |
| *GPER* |  | 10.11 | 0.12 |  |  |  | 15.298 | 0.0181 |  |  |  |
|  | st.16- st.17 |  |  | 1.389 | 0.165 | 0.484 |  |  | 0.205 | 0.837 | 0.837 |
|  | st.16- st.18 |  |  | -0.648 | 0.517 | 0.864 |  |  | 1.847 | 0.065 | 0.233 |
|  | st.16- st.19 |  |  | -0.278 | 0.781 | 0.924 |  |  | -0.656 | 0.512 | 0.597 |
|  | st.16- st.20 |  |  | -0.093 | 0.926 | 0.973 |  |  | -1.231 | 0.218 | 0.458 |
|  | st.16- st.21 |  |  | -1.327 | 0.185 | 0.484 |  |  | 0.821 | 0.412 | 0.54 |
|  | st.16- st.25 |  |  | -0.278 | 0.781 | 0.924 |  |  | 0.937 | 0.349 | 0.54 |
|  | st.17- st.18 |  |  | -2.277 | 0.023 | 0.239 |  |  | 1.835 | 0.066 | 0.233 |
|  | st.17- st.19 |  |  | -1.863 | 0.062 | 0.328 |  |  | -0.923 | 0.356 | 0.54 |
|  | st.17- st.20 |  |  | -1.656 | 0.098 | 0.41 |  |  | -1.606 | 0.108 | 0.289 |
|  | st.17- st.21 |  |  | -3.036 | 0.002 | 0.05 |  |  | 0.688 | 0.491 | 0.597 |
|  | st.17- st.25 |  |  | -1.863 | 0.062 | 0.328 |  |  | 0.821 | 0.412 | 0.54 |
|  | st.18- st.19 |  |  | 0.414 | 0.679 | 0.924 |  |  | -2.565 | 0.01 | 0.108 |
|  | st.18- st.20 |  |  | 0.621 | 0.535 | 0.864 |  |  | -3.441 | 0.001 | 0.012 |
|  | st.18- st.21 |  |  | -0.759 | 0.448 | 0.855 |  |  | -1.147 | 0.251 | 0.48 |
|  | st.18- st.25 |  |  | 0.414 | 0.679 | 0.924 |  |  | -0.821 | 0.412 | 0.54 |
|  | st.19- st.20 |  |  | 0.207 | 0.836 | 0.924 |  |  | -0.513 | 0.608 | 0.672 |
|  | st.19- st.21 |  |  | -1.173 | 0.241 | 0.506 |  |  | 1.539 | 0.124 | 0.289 |
|  | st.19- st.25 |  |  | 0 | 1 | 1 |  |  | 1.592 | 0.111 | 0.289 |
|  | st.20- st.21 |  |  | -1.380 | 0.168 | 0.484 |  |  | 2.294 | 0.022 | 0.126 |
|  | st.20- st.25 |  |  | -0.207 | 0.836 | 0.924 |  |  | 2.257 | 0.024 | 0.126 |
|  | st.21- st.25 |  |  | 1.173 | 0.241 | 0.506 |  |  | 0.205 | 0.837 | 0.837 |

**Table S6 Differences in gene expression at each developmental stage of *GPER* under MPT or FPT**

**Table S7 Differential gene expression of three estrogen receptors under MPT or FPT**

| Treatment | kruskal_statistic | kruskal_p_value | comparison | dunn_z_value | dunn_p_value | *dunn_p_adjusted* |
| --- | --- | --- | --- | --- | --- | --- |
| 26℃/MPT | 41.286 | **0.000** |  |  |  |  |
|  |  |  | ESRα - ESRβ | 2.646 | 0.004 | **0.012** |
|  |  |  | ESRα - GPER | 6.354 | 0.000 | **0.000** |
|  |  |  | ESRβ - GPER | 3.485 | 0.000 | **0.001** |
| 31℃/MPT | 37.527 | **0.000** |  |  |  |  |
|  |  |  | ESRα - ESRβ | 2.576 | 0.005 | **0.015** |
|  |  |  | ESRα - GPER | 6.053 | 0.000 | **0.000** |
|  |  |  | ESRβ - GPER | 3.629 | 0.000 | **0.000** |
